# Fast TV-PRO-seq: Accelerated and Streamlined Protocol for Timing RNA Polymerase Pausing

**DOI:** 10.21769/BioProtoc.5395

**Published:** 2025-07-20

**Authors:** Jie Zhang, Zhixian Liang, Mingxin Sun, Daniel Hebenstreit, Shaohui Zhang

**Affiliations:** 1 National Engineering Research Center for Healthcare Devices & Guangdong Provincial Key Laboratory of Medical Electronic Instruments and Materials, Institute of Biological and Medical Engineering, Guangdong Academy of Sciences, Guangzhou, China; 2 National Engineering Research Center for Healthcare Devices, Institute of Biological and Medical Engineering, Guangdong Academy of Sciences, Guangzhou, China; 3 Guangdong Provincial Key Laboratory of Medical Electronic Instruments and Materials, Institute of Biological and Medical Engineering, Guangdong Academy of Sciences, Guangzhou, China; 4 The College of Life Science and Technology, Jinan University, Guangzhou, China; 5 School of Life Sciences, Gibbet Hill Campus, The University of Warwick, CV4 7AL Coventry, UK

**Keywords:** RNA polymerase pausing, Single-base resolution, Dynamic transcriptional regulation, RNA polymerase II pausing duration, PRO-seq, Nascent transcription, Next-generation sequencing (NGS)

## Abstract

Transcriptional pausing dynamically regulates spatiotemporal gene expression during cellular differentiation, development, and environmental adaptation. Precise measurement of pausing duration, a critical parameter in transcriptional control, has been challenging due to limitations in resolution and confounding factors. We introduce Fast TV-PRO-seq, an optimized protocol built on time-variant precision run-on sequencing (TV-PRO-seq), which enables genome-wide, single-base resolution mapping of RNA polymerase II pausing times. Unlike standard PRO-seq, Fast TV-PRO-seq employs sarkosyl-free biotin-NTP run-on with time gradients and integrates on-bead enzymatic reactions to streamline workflows. Key improvements include (1) reducing experimental time from 4 to 2 days, (2) reducing cell input requirements, and (3) improved process efficiency and simplified command-line operations through the use of bash scripts.

Key features

• Reduces experimental duration from 4 to 2 days via on-bead enzymatic reactions and streamlined workflows.

• Enables single-nucleotide resolution pausing time mapping using time-variant biotin-NTP run-on with saturation kinetics.

• Compatible with reduced cell input (10^6^–10^8^ cells) and sarkosyl-free conditions for improved experimental feasibility.

• Integrates bash scripts and simplified commands for enhanced reproducibility and reduced computational complexity.

## Graphical overview


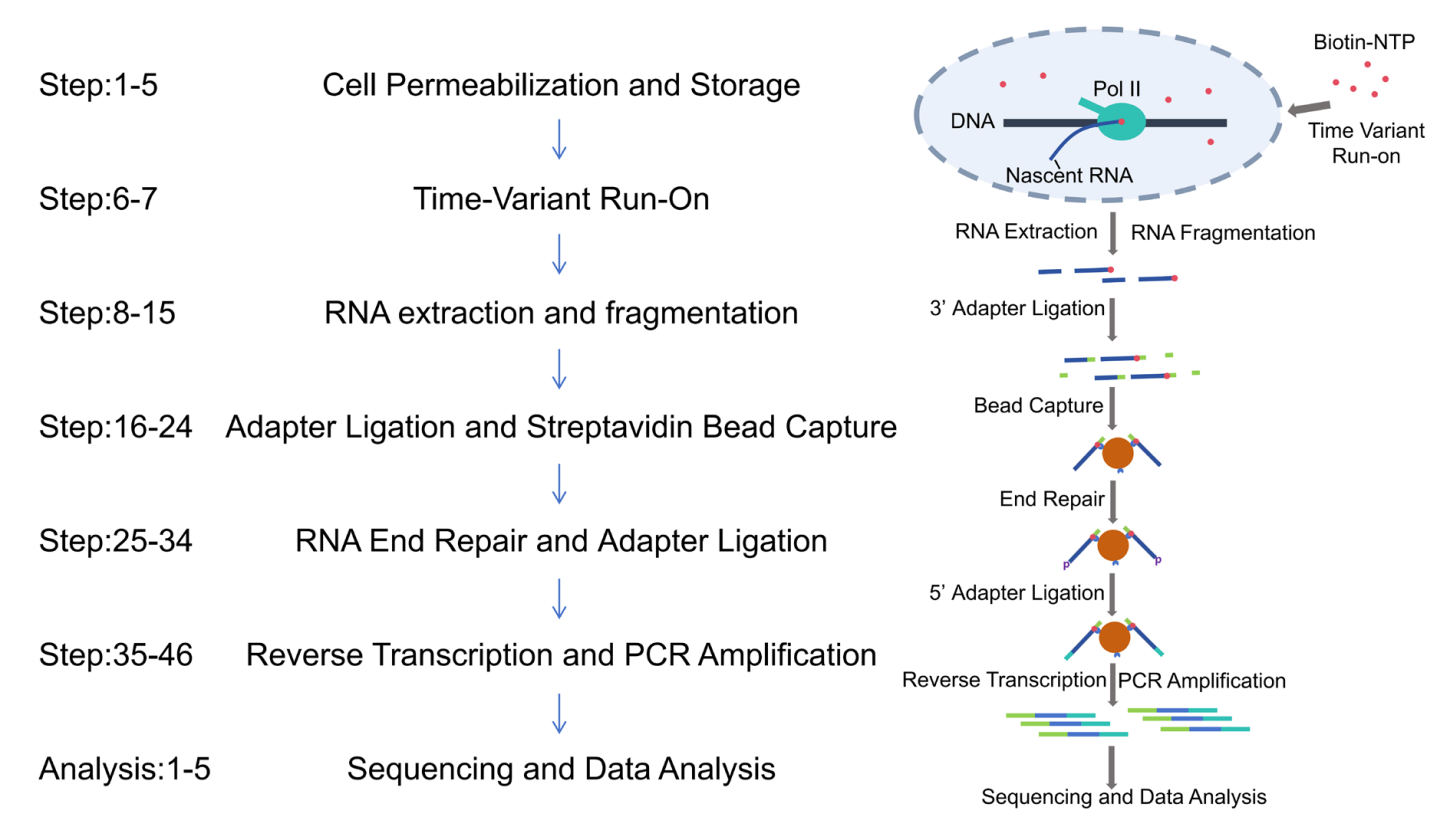


## Background

RNA polymerase II (Pol II) pausing during transcription elongation is a widespread regulatory mechanism in metazoans, particularly in promoter-proximal regions (PPRs) [1,2]. This transient stalling of transcriptionally engaged polymerases influences gene expression dynamics, chromatin states, and transcriptional noise [3,4]. Traditional methods such as Pol II ChIP-seq [5], GRO-seq (global run-on sequencing) [2], NET-seq (native elongating transcript sequencing) [6], mNET-seq [7] (native elongating transcript sequencing technology for mammalian chromatin), and PRO-seq (precision run-on sequencing) have advanced our understanding of polymerase occupancy patterns. However, these approaches primarily measure polymerase occupancy, which conflates pausing time with confounding factors like transcription initiation rates, ratio of pausing, and abortive transcription [8,9]. The widely used triptolide (Trp) treatment method, which inhibits transcription initiation to estimate pause durations in PPRs [3,10], suffers from slow drug uptake and limited resolution [11]. Additionally, newly generated methods highlight that promoter-proximal Pol II accumulation may predominantly arise from high abortive transcription rates, further complicating interpretation [9,12,13].

By performing run-on reactions with varying durations, TV-PRO-seq generates saturation curves that reflect polymerase release kinetics, enabling genome-wide estimation of pausing times at single-nucleotide resolution [14]. This approach bypasses the influence of polymerase turnover and abortive transcription, providing unprecedented insights into the relationship between pausing dynamics, chromatin modifications (e.g., H3K36me3), and transcriptional noise. However, TV-PRO-seq requires the parallel execution of eight sarkosyl-free PRO-seq experiments, a process that is not only time-consuming but also demands substantial cell quantities, ultimately leading to high experimental failure rates that hinder the practical application of TV-PRO-seq. Based on the last version of PRO-seq [15], we present Fast TV-PRO-seq, an enhanced version featuring significantly reduced experimental duration and lower starting cell requirements.

## Materials and reagents

**Reagents**

1. DEPC water (Invitrogen, catalog number: 10514065)

2. NaCl (Sigma-Aldrich, catalog number: S9888)

3. KCl (Sigma-Aldrich, catalog number: P9333)

4. MgCl_2_·6H_2_O (Sigma-Aldrich, catalog number: M2670)

5. 0.5 M EDTA (Sigma-Aldrich, catalog number: 20-158)

6. EGTA (Sigma-Aldrich, catalog number: E3889)

7. Sucrose (Sigma-Aldrich, catalog number: S0389)

8. NaOH (Fisher Chemical, catalog number: 10396240)

9. DTT (Sigma-Aldrich, catalog number: D0632)

10. Glycerol (Sigma-Aldrich, catalog number: G5516)

11. TWEEN-20 (Sigma-Aldrich, catalog number: P9416)

12. Tris-HCl pH 6.8 1 M (AESAR, catalog number: J63831.K2)

13. Tris-HCl pH 7.4 1 M (AESAR, catalog number: J60202.K2)

14. Tris-HCl pH 8.0 1 M (AESAR, catalog number: J62726.K2)

15. 1× PBS pH 7.4 (Gibco, catalog number: 10728775)

16. Ethanol (Fisher Chemical, catalog number: BP2818)

17. Isopropanol (Fisher Chemical, catalog number: BP2816)

18. Chloroform (Fisher Chemical, catalog number: 10488400)

19. Biotin-11-CTP (PerkinElmer, catalog number: NEL542001EA)

20. Biotin-11-UTP (PerkinElmer, catalog number: NEL543001EA)

21. Biotin-11-ATP (PerkinElmer, catalog number: NEL544001EA)

22. Biotin-11-GTP (PerkinElmer, catalog number: NEL545001EA)

23. ATP (New England Biolabs, catalog number: P0756S)

24. GTP (Sigma-Aldrich, catalog number: G3776)

25. Streptavidin Dynabeads C1 (Invitrogen, catalog number: 65002)

26. TRIzol (Invitrogen, catalog number: 15608948)

27. GlycoBlue (Invitrogen, catalog number: 10301575)

28. SUPERase RNase inhibitor (Invitrogen, catalog number: 10773267)

29. T4 RNA ligase I (New England Biolabs, catalog number: M0204)

30. RppH and 10× ThermoPol buffer (New England Biolabs, catalog number: M0356)

31. T4 polynucleotide kinase and 10× PNK buffer (New England Biolabs, catalog number: M0201L)

32. BioZues^®^ III reverse transcriptase (Bioligo, catalog number: SE0301)

33. Q5 master mix (New England Biolabs, catalog number: M0544)

34. AMPure XP beads (Beckman Coulter, catalog number: A63880)

35. Sarkosyl (Sigma-Aldrich, catalog number: L9150)

**Oligos**

3′ RNA adapter: p-GAUCGUCGGACUGUAGAACUCUGAAC -/inverted dT/

STP: CGACGAUCCCACGUUCCCGUGG

5′ RNA adapter: CCUUGGCACCCGAGAAUUCCA

RP1: AATGATACGGCGACCACCGAGATCTACACGTTCAGAGTTCTACAGTCCGA

RPI-n: CAAGCAGAAGACGGCATACGAGATNNNNNNNNGTGACTGGAGTTCCTTGGCACCCGAGAATTCCA (replace NNNNNNNN according to the indexes of Illumina Tru-seq)

**Solutions**

1. Permeabilization buffer (see Recipes)

2. Storage buffer (see Recipes)

3. 1 N NaOH (see Recipes)

4. Washing solution A (see Recipes)

5. Washing solution B (see Recipes)

6. 2× Bead washing buffer (see Recipes)

7. 1× Bead washing buffer (see Recipes)

8. Pre-washed streptavidin beads (see Recipes)

**Recipes**

**1. Permeabilization buffer**

15 mL of 1 M Sucrose, 2.5 mL of 1% Tween-20, 500 µL of 1 M Tris-HCl pH 7.4, 500 µL of 0.1 M EGTA, 500 µL of 10% NP40, 250 µL of 2 M KCI, 250 µL of 1 M MgCl_2_, 25 µL of 1 M DTT, 5 µL of RNase inhibitor, and 1 tablet of protease inhibitors. Add DEPC water up to 50 mL. Make fresh before use.

**2. Storage buffer**

0.2 µL of 0.5 M EDTA, 10 µL of 1 M Tris-HCl pH 8.0, 5 µL of 1 M MgCl_2_, 5 µL of 1 M DTT, 250 µL of glycerol, 730 µL of DEPC water. Make fresh before use.

**3. 1 N NaOH**

2 g of NaOH in 50 mL of DEPC water.

**4. Washing solution A**

100 µL of 1 N NaOH, 10 µL of 5 M NaCl, and 890 µL of DEPC water. Store at 4 °C for 1 month.

**5. Washing solution B**

20 µL of 5 M NaCl and 980 µL of DEPC water. Store at 4 °C for 1 month.

**6. 2× Bead washing buffer**

20 mL of 5 M NaCl, 500 µL of 1 M Tris-HCl pH 7.4, and 5 mL of 1% Tween-20. Add DEPC water up to 50 mL. Store at 4 °C for 1 month.

**7. 1× Bead washing buffer**

25 mL of 2× Bead washing buffer and add DEPC water up to 50 mL. Store at 4 °C for 1 month.

**8. Pre-washed streptavidin beads**

Add 170 µL (20 µL for PRO-seq) Dynabeads MyOne Streptavidin C1 beads in a 1.5 mL tube. Place on a magnetic rack for 2 min and remove the supernatant. Wash beads with 500 µL of washing solution A twice. Wash beads with 500 µL of washing solution B once. Raise the beads in 425 µL (50 µL for PRO-seq) of 2× Bead washing buffer.

**Laboratory supplies**

1. Phase-lock gel tubes (Proandy, catalog number: 10447-1)

2. Low-bind, nuclease-free microcentrifuge tubes (GENEPLASTIX, catalog number: 243-MCT-C-1.5-L)

3. Low-bind, nuclease-free 1,000 µL filtered pipette tips (GENEPLASTIX, catalog number: 222-RF-C-1000S-L)

4. Low-bind, nuclease-free 200 µL filtered pipette tips (GENEPLASTIX, catalog number: 222-RF-C-200S-L)

5. Low-bind, nuclease-free 20 µL filtered pipette tips (GENEPLASTIX, catalog number: 222-RF-C-20S-L)

## Equipment

1. Refrigerated centrifuge (Eppendorf, model: Centrifuge5424R)

2. PCR thermal cycler (LongGene, model: T30D)

3. Heat blocks (JOANLAB, model: MDB100/MDB100-C)

4. Magnetic separator (Invitrogen, model: DynaMag^TM^-2)

5. Rotating stand (BeyoEquip, model: yoVortex^TM^ Basic Rotation Mixer E6800)

## Procedure

1. Collect 10^6^–10^8^ cells (more cells are recommended for a more robust result) with 20 mL of ice-cold PBS into a 50 mL tube. Centrifuge at 1,000× *g* for 5 min at 4 °C.

2. Discard supernatant and resuspend the pellet with 20 mL of ice-cold permeabilization buffer. Centrifuge at 1,000× *g* for 5 min at 4 °C.

3. Discard supernatant and resuspend the pellet with 20 mL of ice-cold permeabilization buffer. Incubate on ice for 10 min.

4. Centrifuge at 1,000× *g* for 5 min at 4 °C, discard supernatant, resuspend the pellet in 500 µL of storage buffer, and transfer the cells into ten 1.5 mL tubes (each with 50 µL).

5. Flash freeze in liquid nitrogen (permeabilized cells can be stored at -80 °C for 1 week).

6. Prepare 2× run-on buffer according to Table 1 and prewarm at 37 °C (see General notes 1 and 2).

**Table 1.** 2× run-on buffer

Reagent	1×	8.5×
1 M MgCl_2_	0.25 µL	2.125 µL
1 M pH 8.0 Tris-HCl	1 µL	8.5 µL
0.1 M DTT	1 µL	8.5 µL
2 M KCl	7.5 µL	63.75 µL
1 mM Biotin-11-CTP	2 µL	17 µL
1 mM Biotin-11-UTP	2 µL	17 µL
1 mM Biotin-11-ATP (ATP)	2 µL	17 µL
1 mM Biotin-11-GTP (GTP)	2 µL	17 µL
2% (w/v) sarkosyl in DEPC water	25 µL	0
DEPC water	6.25 µL	265.625 µL
RNase inhibitor	1 µL	8.5 µL

7. Process run-on reaction according to Table 2 (see General note 3).

**Caution:** The TRIzol^®^ reagent contains phenol and guanidine isothiocyanate, which are highly toxic and corrosive. Always handle it with gloves, eye protection, and under a fume hood.

**Table 2.** Procedure of run-on reaction

Procedure	Time
Preheat stored cells at 37 °C, label the tube as 32-1	0 min
Preheat stored cells at 37 °C, label the tube as 32-2	2 min
Transfer 50 µL of preheated 2× run-on buffer to 32-1, mix by pipetting 15 times	4 min
Transfer 50 µL of preheated 2× run-on buffer to 32-2, mix by pipetting 15 times	6 min
Preheat stored cells at 37 °C, label the tube as 8-1	8 min
Preheat stored cells at 37 °C, label the tube as 8-2	10 min
Transfer 50 µL of preheated 2× run-on buffer to 8-1, mix by pipetting 15 times	12 min
Transfer 50 µL of preheated 2× run-on buffer to 8-2, mix by pipetting 15 times	14 min
Add 500 µL of TRIzol to 8-1, vortex briefly, and place on ice	20 min
Add 500 µL of TRIzol to 8-2, vortex briefly, and place on ice	22 min
Preheat stored cells at 37 °C, label the tube as 2-1	24 min
Transfer 50 µL of preheated 2× run-on buffer to 2-1, mix by pipetting 15 times	28 min
Add 500 µL of TRIzol to 2-1, vortex briefly, and place on ice	30 min
Add 500 µL of TRIzol to 32-1, vortex briefly, and place on ice	36 min
Add 500 µL of TRIzol to 32-2, vortex briefly, and place on ice	38 min
Preheat stored cells at 37 °C, label the tube as 2-2	40 min
Transfer 50 µL of preheated 2× run-on buffer to 2-2, mix by pipetting 15 times	44 min
Add 500 µL of TRIzol to 2-2, vortex briefly, and place on ice	46 min
Preheat stored cells at 37 °C, label the tube as 0.5-1	48 min
Transfer 50 µL of preheated 2× run-on buffer to 0.5-1, mix by pipetting 15 times	52 min
Add 500 µL of TRIzol to 0.5-1, vortex briefly, and place on ice	52.5 min
Preheat stored cells at 37 °C, label the tube as 0.5-2	54 min
Transfer 50 µL of preheated 2× run-on buffer to 0.5-2, mix by pipetting 15 times	58 min
Add 500 µL of TRIzol to 0.5-2, vortex briefly, and place on ice	58.5 min

8. Incubate all samples at room temperature for 10 min.

9. Add 130 µL of chloroform and incubate at room temperature for 5 min. Vortex vigorously for 15 s.

10. Transfer the mixture to 1.5 mL phase-lock gel tubes and incubate at room temperature for 5 min. (Phase-lock gel tubes are optional but recommended to simplify phase separation during RNA extraction.)

11. Centrifuge at 14,000× *g* for 2 min at 4 °C and transfer supernatant to new 1.5 mL tubes.

12. Add 1 µL of GlycoBlue and an equal volume of isopropanol. Vortex and incubate at room temperature for 10 min.

13. Centrifuge at 14,000× *g* for 20 min at 4 °C. Discard supernatant and dissolve RNA pellet in 30 µL of DEPC water.

14. Place the RNA solution on ice, add 7.5 µL of 1 N NaOH, and incubate on ice for 10 min.

15. Add 37.5 µL of pH 6.8 Tris-HCl buffer, 125 µL of DEPC water, 1 µL of GlycoBlue, 8 µL of 5 M NaCl, and 0.5 mL of ice-cold ethanol. Vortex and centrifuge at 16,000× *g* for 30 min at 4 °C.

16. Discard supernatant and dissolve RNA in 6 µL of DEPC water, add 1 µL of 20 µM 3′ RNA adapter, denature at 65 °C for 40 s, and immediately cool on ice.

17. Add 13 µL of ligation mix to RNA as shown in Table 3, mix thoroughly, and incubate at 25 °C for 1 h. (For more complete ligation reactions, the reaction can be extended to 20 °C for 4 h.)

**Table 3.** Ligation mix

Reagent	1×	8.5×
10× T4 RNA ligase buffer	2 µL	17 µL
ATP (10 mM)	2 µL	17 µL
RNase inhibitor	1 µL	8.5 µL
50% PEG8000	6 µL	51 µL
T4 RNA ligase I	2 µL	17 µL

18. Add 30 µL of DEPC water and 50 µL of pre-washed streptavidin beads. Incubate at room temperature for 30 min with 10 rpm rotation.

19. Place tubes on a magnetic rack, wait for 2 min, and discard supernatant.

20. Wash beads with 500 µL of 1× bead washing buffer and discard supernatant.

21. Mix 5 µL of 100 µmol/L STP with 170 µL of DEPC water (20 µL for basic PRO-seq), denature at 95 °C for 30 s, and immediately cool on ice.

22. Add 20 µL of denatured STP to the beads and incubate at room temperature for 10 min with 10 rpm rotation.

23. Place tubes on a magnetic rack, wait for 2 min, and discard supernatant.

24. Wash beads with 500 µL of 1× bead washing buffer and discard supernatant.

25. Discard supernatant using a magnetic rack, resuspend beads in 20 µL of RppH mix as shown in Table 4, and incubate at 37 °C for 30 min.

**Table 4.** RppH mix

Reagent	1×	8.5×
DEPC water	16 µL	136 µL
10× ThermoPol buffer	2 µL	17 µL
RppH	1 µL	8.5 µL
SUPERase-IN RNase inhibitor	1 µL	8.5 µL

26. Place tubes on a magnetic rack, wait for 2 min, and discard supernatant.

27. Wash beads with 500 µL of 1× bead washing buffer and discard supernatant.

28. Discard supernatant, resuspend beads in 20 µL of PNK mix as in Table 5, and incubate at 37 °C for 30 min.

**Table 5.** PNK mix

Reagent	1×	8.5×
DEPC water	14 µL	119 µL
10× PNK buffer	2 µL	17 µL
10 mM ATP	2 µL	17 µL
T4 PNK (NEB)	1 µL	8.5 µL
SUPERase-IN RNase Inhibitor	1 µL	8.5 µL

29. Place tubes on a magnetic rack, wait for 2 min, and discard supernatant.

30. Wash beads with 500 µL of 1× bead washing buffer and discard supernatant.

31. Resuspend beads in 20 µL of ligation mix as shown in Table 6 and incubate at 25 °C for 1 h. (For more complete ligation reactions, the reaction can be extended to 20 °C for 4 h.)

**Table 6.** 5’ adapter ligation mix

Reagent	1×	8.5×
10× T4 RNA ligase buffer	2 µL	17 µL
ATP (10 mM)	2 µL	17 µL
RNase inhibitor	1 µL	8.5 µL
50% PEG8000	6 µL	51 µL
T4 RNA ligase I	2 µL	17 µL
DEPC water	6 µL	51 µL
20 μM 5′ RNA adapter	1 µL	8.5 µL

32. Place tubes on a magnetic rack, wait for 2 min, and discard supernatant.

33. Wash beads with 500 µL of 1× bead washing buffer and discard supernatant.

34. Repeat step 33.

35. Add 1 µL of 20 μM RP1 primer, denature at 95 °C for 30 s, and immediately cool on ice.

36. Add 17 µL of reverse transcription premix (BioZues^®^ III reverse transcriptase may be replaced by other high-fidelity reverse transcriptase) (Table 7):

**Table 7.** Reverse transcription premix

Reagent	1×	8.5×
2× BioZues III RT buffer	10 µL	85 µL
DEPC water	7 µL	68 µL

37. Add 1 µL of BioZues III RT enzyme and 1 µL of RNase inhibitor and transfer the mixture to a PCR tube.

38. Run program on a PCR machine: 50 °C for 10 min, 55 °C for 10 min, and 4 °C hold.

39. Add 31 µL of NEBNext Ultra II Q5 Master Mix, 10 µL of DEPC, and 1 µL of 20 μM RPI-n primer.

40. Run program on a PCR machine: 95 °C for 10 min; 5 cycles of (95 °C 30 s, 56 °C 30 s, 72 °C 30 s); 10 cycles of (95 °C 30 s, 65 °C 30 s, 72 °C 30 s); 72 °C for 10 min; and 4 °C hold.

41. Add 110 µL of room temperature AMPure XP beads to the PCR production and transfer the mixture to a 1.5 mL tube. Incubate at room temperature for 5 min (see Troubleshooting 1).

42. Place tubes on a magnetic rack, wait for 2 min, and discard supernatant.

43. Wash the beads twice with 70% ethanol without resuspending.

44. Discard supernatant and air dry the beads for 5 min.

45. Resuspend beads in 22 µL of 10 mM Tris-Cl, pH 8.0, and incubate at room temperature for 5 min.

46. Place the beads on a magnet stand and transfer supernatant to a new tube for sequencing.

## Data analysis

1. Script is summarized at https://github.com/zhangjie-sequencing/Fast-TV-PRO-seq.git .

2. Move all the .*fastq/.fastq.gz* files into a new folder and download the *PRO_raw_processing.sh* script from GitHub to the same folder. Change the BOWTIE2_INDEX with your path, then directly run:

./PRO_raw_processing.sh

The versions of the bioinformatics tools used in this protocol are presented in Table 8.

**Table 8.** Bioinformatics tools

Bioinformatic tools	Version
cutadapt	4.9
bowtie2	2.5.4
samtools	1.21
bedtools	2.31.1

3. Process the .fastq/.fastq.gz file to a bedgraph file (see Troubleshooting 2). Merge the bedgraph files into two files and arrange the bedgraph file as follows:

perl bedgraph_merge.pl 0.5_1_p.bedgraph 0.5_2_p.bedgraph 2_1_p.bedgraph  2_2_p.bedgraph 8_1_p.bedgraph 8_2_p.bedgraph 32_1_p.bedgraph 32_2_p.bedgraph > merge_p.bedgraph

perl bedgraph_merge.pl 0.5_1_m.bedgraph 0.5_2_m.bedgraph 2_1_m.bedgraph  2_2_m.bedgraph 8_1_m.bedgraph 8_2_m.bedgraph 32_1_m.bedgraph 32_2_m.bedgraph > merge_m.bedgraph

4. Perform peak calling as follows:

perl TV_callpeak.pl merge_p.bedGraph merge_m.bedGraph

This script will generate two files: the *TVPRO_peak* file records the original reads of all peaks [14], and the *chr_read* file records the total reads of the mitochondrion and nucleus (see Troubleshooting 3).

5. Generate the final result using the following command line:

python average_time_count.py

The final pausing time estimation is inside the file *pausing_times* (see General note 4).

## Validation of protocol

This protocol has been used and validated in the following research article:

• Jie Zhang et al. [14]. TV-PRO-seq reveals the pausing time of pausing sites at base pair resolution.

**Figure 1.**
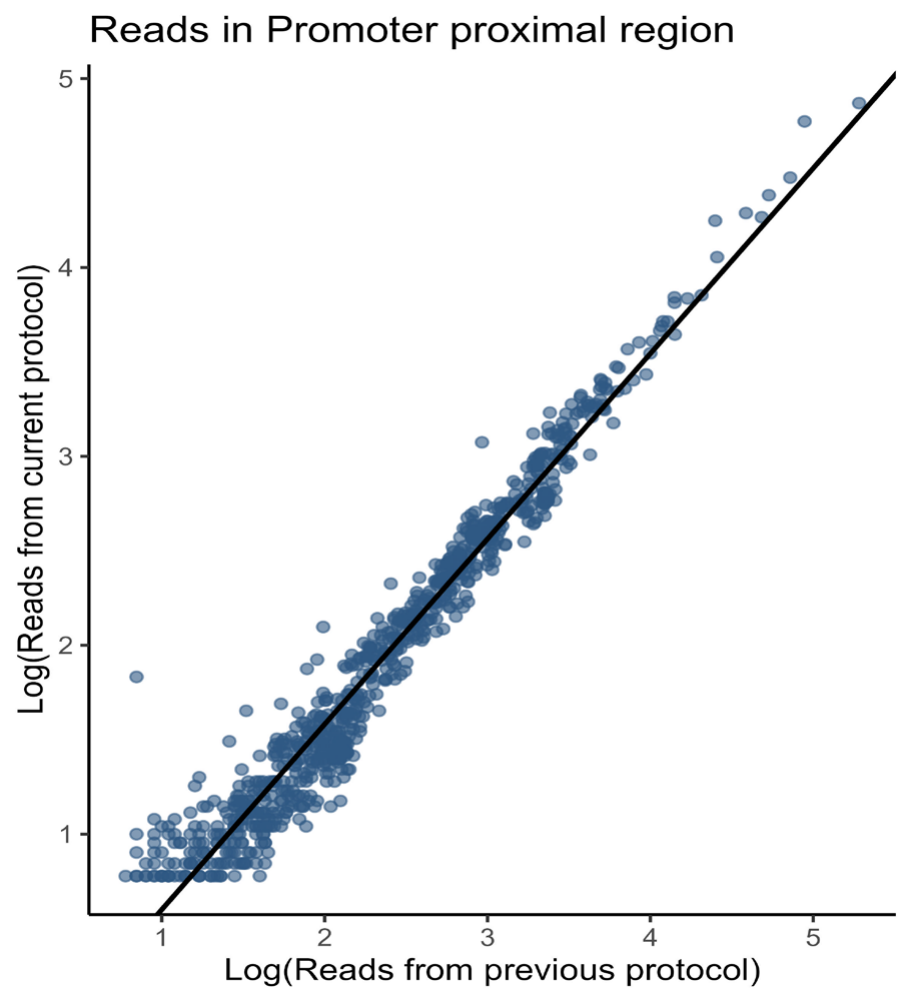
Fast TV-PRO-seq shows a strong correlation with the original TV-PRO-seq [14]. Comparison of promoter-proximal (TSS to +100 bp) read counts between original TV-PRO-seq (X-axis) and Fast TV-PRO-seq (Y-axis). The read counts exhibit a high degree of concordance between the two methods, as evidenced by a Pearson correlation coefficient of 0.97.

This protocol is an enhanced version of our previous TV-PRO-seq (PRO-seq) protocol [14]. The two versions show a correlation exceeding 97% in the promoter-proximal regions of genes (Figure 1).

## General notes and troubleshooting

**General notes**

1. The "1×" condition corresponds to the basic PRO-seq library preparation protocol, while "8.5×" refers to the scaled-up TV-PRO-seq method. When using the TV-PRO-seq method, the same procedure should be applied to all eight samples after the run-on step.

2. If budget constraints exist, ATP and GTP can be used as alternatives for biotin-11-ATP and biotin-11-GTP in the run-on reaction.

3. The TV-PRO-seq experiment includes four distinct run-on time points: 0.5, 2, 8, and 32 minutes, each with two biological replicates. The tube labels follow the pattern [Time]-[Replicate]. The timetable in this protocol is for reference only. The experimental sequence can be flexibly arranged. However, it is essential to ensure that each of the run-on time points (0.5 min, 2 min, 8 min, and 32 min) has two replicates.

4. TV-PRO-seq estimates transcription pause durations via kinetic modeling, and absolute time values should be interpreted as relative measures. Direct quantitative comparisons between samples are discouraged due to potential batch effects. For cross-sample analyses, we recommend using rank-based normalization or selecting a subset of stable ("housekeeping") paused peaks as internal controls.

**Troubleshooting**

1. If high adapter dimmer contamination exists, PAGE gel purification can replace AMPure bead-based purification following the protocol of PRO-seq [15].

2. You can also use a step-by-step command line instead of PRO_raw_processing.sh:

Remove adapter: cutadapt -m 20 -e 0.05 -a TGGAATTCTCGGGTGCCAAGG

Alignment: bowtie2 -k 1 --very-sensitive --no-unal -x -U

Sam to Bam: samtools view -bS

Sort bam file: samtools sort

Plus strand read: bedtools genomecov -strand - -5 -bga -ibam .sorted.bam > _p.bedgraph

Minus strand read: bedtools genomecov -strand + -5 -bga -ibam .sorted.bam > _m.bedgraph

3. The analysis script requires mitochondrial chromosomes to be labeled as chrM, M, or MtDNA in the first column of the bedgraph files. If your reference genome uses alternative nomenclature (e.g., mitochondrion), rename the chromosome in the input files prior to analysis.
